# More than 20 years after re-emerging in the 1990s, diphtheria remains a public health problem in Latvia

**DOI:** 10.2807/1560-7917.ES.2016.21.48.30414

**Published:** 2016-12-01

**Authors:** Ieva Kantsone, Irina Lucenko, Jurijs Perevoscikovs

**Affiliations:** 1 European Programme for Intervention Epidemiology Training (EPIET), European Centre for Disease Prevention and Control, Stockholm, Sweden; 2 Infectious Diseases Surveillance and Immunisation Unit, Infectious Disease Risk Analysis and Prevention Department, Centre for Disease Prevention and Control, Riga, Latvia; 3 Infectious Disease Risk Analysis and Prevention Department, Centre for Disease Prevention and Control, Riga, Latvia

**Keywords:** Diphtheria, surveillance, bacterial infections, vaccine-preventable diseases

## Abstract

In 1994, the World Health Organization (WHO) declared the goal of eliminating diphtheria within the WHO European Region by the year 2000. However, in 1990 an epidemic emerged within the Russian Federation and spread to other countries, including Latvia, by 1994. We describe national surveillance and immunisation coverage data in Latvia from 1994 to 2014 and present historical data from 1946. We defined a laboratory-confirmed case as a clinical case in which toxin-producing *Corynebacterium diphtheriae*, *C. ulcerans* or *C. pseudotuberculosis* was isolated. From 1994 to 2014, 1,515 cases were reported, giving an average annual incidence of 3.2 cases per 100,000 inhabitants (range 0.1–14.8), with the highest incidence in age groups 5–19 and 40–49 years (4.4 and 4.3/100,000, respectively); 111 deaths were reported, 83.8% cases were laboratory-confirmed. Most cases occurred in unvaccinated adults. To improve disease control a supplementary immunisation campaign for adults was initiated in 1995, and by the end of 1998 national coverage among adults reached 70%, and reached 77% in 2003, but declined to 59% by 2014. Diphtheria remains a problem in Latvia with continued circulation of toxin-producing strains of *C. diphtheriae*. We recommend to strengthen immunisation to cover adults, as well as the education of health professionals and a serological survey.

## Introduction

Diphtheria is a highly contagious communicable disease caused by toxin-producing strains of *Corynebacterium diphtheriae* (or rarely by *Corynebacterium ulcerans* or *Corynebacterium pseudotuberculosis*) and transmitted through respiratory droplets during close contact, primarily infecting the pharynx, tonsils and nose. Diphtheria toxin is absorbed at the site of the lesions and may affect other organs far from the initial area of infection, such as the heart, nervous system, and kidneys. Diphtheria antitoxin is the specific treatment for diphtheria and must be given immediately when clinicians suspect a diphtheria case. Successful treatment of diphtheria depends on rapid administration of equine diphtheria antitoxin in combination with antibiotics [1]. Diphtheria can be prevented by vaccination.

Many countries have progressed towards the elimination of diphtheria. However, inadequate healthcare delivery systems, poverty and other social factors have led to diphtheria re-emerging and remaining endemic in many regions of the world [2]. Diphtheria still circulates in several countries in Africa, the eastern Mediterranean, eastern Europe, South America, south-east Asia and the South Pacific [3,4].

It was thought that indigenous diphtheria would be eliminated within the World Health Organization (WHO) European Region by the year 2000 following the success of the mass immunisation programme introduced more than 60 years ago [5,6]. In 1994, the WHO European Region proposed elimination of indigenous diphtheria by the year 2000 [6]. However, an epidemic had already emerged in 1990 in the Russian Federation and from 1991 to 1993 spread to neighbouring countries [7,8]. Although the affected countries succeeded in reducing diphtheria incidence, diphtheria remained endemic in Belarus, Georgia, Latvia, the Russian Federation and Ukraine. Other European countries reported sporadic imported cases between the years 2000 and 2013 (Belgium, Estonia, Finland, France, Germany, Lithuania, Netherlands, Sweden, UK) [3,9].

Despite the fact that diphtheria is a somewhat forgotten disease in many European countries, it remains a serious health problem in endemic countries and a potential threat for other countries considered to be disease-free. More recently, awareness has increased due to several sporadic cases being reported in Europe, and in particular a recent fatal case in Spain and cutaneous diphtheria cases in refugees and asylum seekers in Denmark, Germany and Sweden; the issue of shortages of diphtheria antitoxin was also highlighted as an European Union priority [10,11].

In 1994, an epidemic started in the Baltic States, and Latvia was the most affected of these three countries [12]. The supplementary immunisation campaign initiated in 1995 led to improved disease control but vaccination coverage was not sufficient for eradication [13]. Between 1999 and 2014, Latvia reported the highest annual incidence of diphtheria in the WHO European Region [3].

Here, we describe trends over time based on national surveillance data and data on immunisation coverage from 1994 to 2014 in Latvia, complemented by historical data since 1946, to provide insight into the epidemiology of diphtheria more than 20 years after its re-emergence and to better target future prevention strategies.

## Methods

Our study period is from 1994 to 2014, and we also describe historical data from 1946 onwards. We obtained and analysed national surveillance data. From 1946 to 2001 data was available in aggregated form and case-based data were available from 2002 to 2014.

### Case definition

The case definition used for surveillance of diphtheria has changed between 1994 and 2014. Since 2002, we have used the European Union case definition for reporting to the Community network [14]. Cases included in annual reports before 2002 did not use a standardised case definition. In this paper, we analysed all reported clinically and/or laboratory-confirmed cases included in our annual statistical reports from 1994 to 2014. For our study we defined a clinically confirmed or suspected case as diagnosed by a physician with a typical clinical picture, e.g. upper respiratory tract illness with laryngitis or nasopharyngitis or tonsillitis with or without an adherent membrane/pseudomembrane, and for cutaneous diphtheria skin lesion diphtheria of other sites - conjunctiva or mucous membranes. We defined a laboratory-confirmed case as a case with clinical picture and the isolation of toxin-producing *C. diphtheriae*, *C. ulcerans* or *C. pseudotuberculosis* from a clinical specimen.

### Case and contact management

According to the Latvian procedures for registration of infectious diseases, all cases, suspected and confirmed, should be notified within 1 working day to the local public health structure [15]. Physicians should take swabs to confirm the diagnosis before antibiotic treatment is started. Depending on the clinical condition of the patient, diphtheria antitoxin may be given. Patients should be immunised in the convalescent stage.

Swabs should be taken from all close contacts, who should be provided with prophylactic antibiotics and monitored daily for at least 7 days. Immunisation should be offered if contacts have not been vaccinated [16].

### Description of surveillance

During the study period, physicians notified all suspected cases of diphtheria to local public health structure using standardised notification forms according to the Regulations of the Cabinet of Ministers of Latvia valid at the time of reporting [17].

Following notification, the local epidemiologist began the investigation using a dedicated case investigation form and after completion, submitted this to the national level using electronic surveillance system. This case investigation form included information on clinical signs and symptoms, outcome, complications, laboratory data, vaccination status, history of travel, management of the case and contacts, etc.

### Vaccination status

Regional epidemiologists ascertained vaccination status by checking patients’ medical cards. This ascertainment took into account that in the first year of life children should receive the primary three-dose immunisation course of diphtheria vaccination. By the age of 12 months to 15 years, children should have received an additional three booster doses. It is recommended that adults over the age of 25 years have a booster dose every 10 years, free of charge. If more than 10 years had elapsed since the last booster dose, two doses of tetanus–diphtheria (Td) vaccine were recommended (the second dose administered at 4–6 weeks after the first dose).

An unvaccinated adult was defined as an individual who had not previously been immunised against diphtheria, had not received a booster vaccination for more than 10 years or whose vaccine status was unknown [18]. A partially vaccinated individual was defined as a person who had started vaccination and received at least one dose of vaccine against diphtheria, but missed one or more doses of primary immunisation or booster dose for children or the second booster dose for adults (i.e. when an adult had received the most recent booster dose more than 10 years ago).

There were only slight changes in the Latvian immunisation programme between 1994 and 2014 (Table 1) [18,19].

**Table 1:** Diphtheria immunisation programme in Latvia, 1994–2014

Immunisation dose	Age of immunisation		
	1994–1997	1998–2008	2009–2014
**1st dose**	3 months	3 months	2 months
**2nd dose**	4.5 months	4.5 months	4 months
**3rd dose**	6 months	6 months	6 months
**1st booster dose**	18 months	18 months	12–15 months
**2nd booster dose**	9 years	7 years	7 years
**3rd booster dose**	15–16 years	14 years	14 years
**Adult booster dose^a^**	Every 10 years, starting at the age of 25 years		

^a^ If more than 10 years have elapsed since the last booster dose, two doses of vaccine are recommended, with the second dose given 4–6 weeks after the first dose.

### Severity of disease

Symptoms of diphtheria can vary from mild to severe. Physicians defined severity of disease according to the distribution of the membrane and severity of symptoms of intoxication. Mild disease was defined as localised (affects only the nose, tonsils, or nose and throat) and moderate disease as a case with a more widely distributed membrane (affecting the nose, tonsils, throat and the entire tracheobronchial tree). Severe disease was defined as a case with widely distributed membrane and severe intoxication and/or systemic complications (myocarditis, neuritis and other systemic toxic effects) and/or death.

### Laboratory investigation

Clinical specimens were taken from suspected diphtheria cases by clinicians for microbiological analysis (isolation and toxigenicity testing). All private and hospital laboratories in Latvia submitted cultures to the national reference laboratory for identification and toxigenicity testing.

### Immunisation coverage

Immunisation coverage in children was routinely determined for each dose of vaccine by the National Public Health Institute. For the numerator, we used the number of vaccine doses administered by vaccination services annually, based on monthly reports. For the denominator, we used population estimates from the Central Statistical Bureau of Latvia [20].

The Institute also measured vaccination coverage among adults. To assess vaccination coverage among adults aged ≥ 25 years we divided the number of adults who received a third dose (of the primary three-dose immunisation course) or booster dose in the previous 10 years in the age group ≥ 25 years by the number of adults in that age group at the beginning of the reference year.

### Statistical analysis

To describe trends over time and to provide the current epidemiology of diphtheria, existing surveillance data was summarised. We analysed cases’ vaccination status and age with clinical presentation of disease in terms of the frequency of severity of disease.

Categorical variables were summarised using frequencies and proportions. To calculate the incidence, the resident population estimates for each year obtained from the Central Statistical Bureau of Latvia were used [20]. There have been changes over time among the Latvian population due to emigration, low birth rate and other factors. The population shrank from 2.5 million inhabitants at the beginning of 1994 to 2.0 million at the beginning of 2014 [20].

## Results

### Historical trends in Latvia

At the end of the 1940s, diphtheria incidence was very high, reaching 108.9 per 100,000 inhabitants in 1946. From 1968 to 1985, no diphtheria cases were reported but there were 51 cases registered from 1986 to 1993 (Figure).

**Figure:**
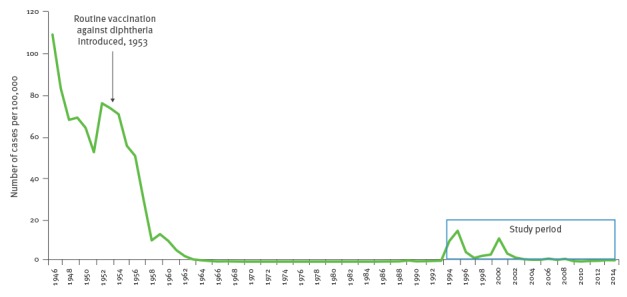
Incidence rate of diphtheria in Latvia, 1946–2014

There was a sharp increase in incidence from 0.5 per 100,000 inhabitants in 1993 to 14.8 per 100,000 inhabitants in 1995. The incidence decreased by 1996 to 4.5 per 100,000 inhabitants. A second wave of increasing incidence was observed in 2000 (11.1/100,000). In 2000, an outbreak occurred among highly vaccinated trainees at a Latvian military academy; 45 cases were identified [21].

### Cases from 1994 to 2014

From the beginning of the epidemic in 1994 to 2014, 1,515 cases were reported, giving an average annual incidence of 3.2 cases per 100,000 inhabitants (range: 0.1/100,000 (2010) to 14.8/100,000 (1995)).

#### Age and sex distribution

Of these cases, 56.3% were female and 43.7% male. The highest incidence was in the age groups 5–19 and 40–49 years (4.4 and 4.3/100,000, respectively) (Table 2). Compared with 2009–2011, in 2012–2014 more cases were recorded among persons aged under 18 years.

**Table 2:** Reported number of cases and rates of diphtheria by age, sex, and case fatality rate, Latvia, 1994–2014

		Number of cases	Proportion of the total	Cumulative incidence per 100,000	Deaths	Proportion of the total	Case fatality rate (%)
**Age group (years)**	**0–4**	84	5.5%	3.6	11	9.9%	13.1
	**5–19**	396	26.1%	4.4	3	2.7%	0.8
	**20–29**	195	12.9%	2.9	1	0.9%	0.5
	**30–39**	192	12.7%	2.9	9	8.1%	4.7
	**40–49**	286	18.9%	4.3	37	33.3%	12.9
	**50–59**	218	14.4%	3.6	29	26.1%	13.3
	**≥ 60**	144	9.5%	1.4	21	18.9%	14.6
**Sex**	**Males**	662	43.7%	3.0	NA	NA	NA
	**Females**	853	56.3%	3.3	NA	NA	NA
**Vaccination status**	**Fully vaccinated**	418	27.6%	NA	1	NA	0.2
	**Partially vaccinated**	67	4.4%	NA	NA	NA	NA
	**Un-vaccinated**	1,030	68.0%	NA	110	NA	10.0
**Total**		1,515	100%	3.2	111	100%	7.3

NA: not available.

### Vaccination status

Of 1,515 cases, 68.0% were unvaccinated, 4.4% were partially vaccinated and 27.6% were fully vaccinated. Of all fatal cases (n = 111) only one was fully vaccinated and the remainder were unvaccinated.

### Outcome and severity of disease

A total of 111 deaths were reported, of which 33.3% were in the age group 40–49 years. The case fatality rate was 7.3%, varying from 0.5% to 14.6% in different age groups. The highest case fatality rate was among adults in the age group ≥ 60 years (14.6%), 50–59 years (13.3%) and among children under 5 years (13.1%) (Table 2).

Among reported cases, 21.0% were ascertained as severe, 47.5% as moderate and 31.5% as mild (Table 3). Severe forms of disease represented 23.4% of diphtheria cases among adults and 12.6% among children aged 0–17 years. Of all cases with severe form of disease 93.7% were partially vaccinated or unvaccinated and 6.3% vaccinated.

**Table 3:** Proportion of diphtheria cases by severity of disease and vaccination status in Latvia, 1994–2014

Severity of disease		Number of severe cases	%	Number of moderate cases	%	Number of mild cases	%
**Age group (years)**	**0–4**	22	28.9%	25	32.9%	29	38.2%
	**5–9**	14	12.1%	33	28.4%	69	59.5%
	**10–14**	3	3.2%	30	32.3%	60	64.5%
	**15–17**	4	7.1%	20	35.7%	32	57.1%
	**18–19**	5	3.6%	104	74.3%	31	22.1%
	**20–29**	5	2.7%	99	52.7%	84	44.7%
	**30–39**	26	13.2%	91	46.2%	80	40.6%
	**40–49**	95	33.9%	138	49.3%	47	16.8%
	**50–59**	81	37.3%	105	48.4%	31	14.3%
	**≥ 60**	63	41.4%	74	48.7%	15	9.9%
**Children 0–17 years**		**43**	**12.6%**	**108**	**31.7%**	**190**	**55.7%**
**Adults ≥ 18 years**		**275**	**23.4%**	**611**	**52.0%**	**288**	**24.5%**
**Vaccination status**	**Vaccinated**	20	4.8%	203	48.6%	195	46.6%
	**Partially vaccinated or unvaccinated**	298	27.2%	516	47.0%	283	25.8%
**Total**		**318**	**21.0%**	**719**	**47.5%**	**478**	**31.5%**

During 2002–2014 when case-based data were available, 59.4% of all cases (142/239) and 17 of 20 fatal cases received diphtheria antitoxin.

### Laboratory investigations

From 1994 to 2014, 83.8% of all cases (1,270/1,515) were laboratory confirmed. Of these *C. diphtheria* cases, 92.4% had biovar gravis and 5.2% were biovar mitis. A toxigenic strain of *C. ulcerans* was identified only from one case in 2009. Biovar gravis was prevalent during the epidemic period. Although in the pre-epidemic period 1986–1993 biovar mitis dominated; 54.1% of strains identified were biovar mitis, and 45.9% of strains were biovar gravis.

### Seasonality

More cases had their onset of symptoms during the autumn (September, October, November; n = 583; 38.5%), but between other seasons there were no apparent differences.

### Immunisation programme

Childhood vaccination coverage with three, five or six doses of diphtheria vaccine fell from 1989 to 1995.

Mass immunisation of adults was initiated in 1995. By the end of 1998 the national coverage among adults was 70%. The immunisation programme achieved high national vaccination coverage for adults of 77%, in 2003 but it deteriorated to 59% in 2014.

From 2000 to 2014, childhood vaccination coverage with a third dose ranged from 91% to 98% and with a fifth dose from 92% to 98%. From 2000 to 2014, vaccination coverage for adolescents (sixth dose at 15 years) ranged from 86% to 96%, decrease in coverage occurred from 96% in 2007 to 86% in 2014.

## Discussion

Starting from 1994 Latvia experienced an increase in diphtheria cases, and during 1999–2014, Latvia reported the highest annual incidence of diphtheria within the EU and in the WHO European Region [3]. Although in European countries diphtheria is an uncommon disease, it is still endemic in Latvia [22]. Despite high vaccination coverage, incidence increased from 0.1 per 100,000 inhabitants in 2010 to 0.7 in 2013. The highest incidence was among the age groups 5–19 and 40–49 years. No cases in children were observed from 2009 to 2011, but new cases have emerged since 2012.

Most cases occurred in adults who were either unvaccinated or incompletely vaccinated, and these subgroups had the most severe outcomes. The proportion of severe forms was six times higher among those who were unvaccinated of partly vaccinated in comparison to those who were fully vaccinated. Only 4.3% of vaccinated cases had the severe form of diphtheria and one case was fatal. This indicated that the disease in vaccinated individuals was milder and less fatal. The case fatality rate in the unvaccinated was more than 50 times higher compared with those vaccinated (10.0% vs 0.2%). The highest case fatality rate was among adults in the age groups ≥ 50 years; and among children under 5 years old. These population groups, children and older adults who did not have up-to-date immunisations, were defined as the high-risk groups [23]. From 1996 to 2003 annual seroepidemiological studies were carried out in Latvia. Studies in European countries have indicated that immunity levels below the protective level (> 0.1 IU) increased with age of adults [24]. On average in Latvia, for 23% of adults the immunity level was lower than protective and for 30% of adults it was protective. The highest number of seronegative adults was detected in adults aged ≥ 50 years. This may explain the large number of severe cases and high morbidity and mortality rate among adults over 50 years old.

Our investigation had some limitations. A lack of case-based data before 2002 required us to limit the scope of our analysis. Misclassification of vaccination status may have occurred due to poor documentation of vaccinations and this may have led to an overestimating of the rate of unvaccinated individuals.

Diphtheria remains a public health problem in Latvia with continued circulation of toxin-producing stains of *C. diphtheriae*. Maintaining high vaccination coverage is essential to prevent the re-emergence of *C. diphtheriae*. This was exemplified by the re-emergence of diphtheria parallel with a decline of childhood vaccination coverage with three doses of vaccine during the first year of life from 90% in 1989 to 77% in 1995, and for the fifth dose at the age of 9 years from 97% to 90%, and for the sixth dose at the age 15 years from 98% to 80% [13]. This supports the WHO recommendation of achieving vaccination coverage above 90% for children and at least 75% for the adult population to eliminate the disease [6]. According to the goals of the national public health strategy for 2014 to 2020 we should achieve vaccination coverage for at least 95% of children and at least 62–65% of the adult population in Latvia [25].

The National Public Health Institute recommends to strengthen immunisation to cover adults with adequate booster dose(s) or three doses and continuous education of health professionals on how to talk with patients about their concerns of vaccines. We also suggest conducting a serological survey to document the current immunity to diphtheria.
